# Food waste conversion to microbial polyhydroxyalkanoates

**DOI:** 10.1111/1751-7915.12776

**Published:** 2017-07-24

**Authors:** Chad Nielsen, Asif Rahman, Asad Ur Rehman, Marie K. Walsh, Charles D. Miller

**Affiliations:** ^1^ Department of Biological Engineering Utah State University 4105 Old Main Hill Logan UT 84322‐4105 USA; ^2^ Bioengineering Branch Space BioSciences Division NASA Ames Research Center Moffett Field CA 94035‐1000 USA; ^3^ COSMIAC Research Center University of New Mexico Albuquerque NM 87106 USA; ^4^ Institute of Industrial Biotechnology Government College University Katchery Road Lahore Pakistan; ^5^ Department of Nutrition, Dietetics, and Food Sciences Utah State University 8700 Old Main Hill Logan UT 84322‐8700 USA

## Abstract

Polyhydroxyalkanoates (PHAs) are biopolymers with desirable material properties similar to petrochemically derived plastics. PHAs are naturally produced by a wide range of microorganisms as a carbon storage mechanism and can accumulate to significantly high levels. PHAs are an environmentally friendly alternative to their petroleum counterparts because they can be easily degraded, potentially reducing the burden on municipal waste systems. Nevertheless, widespread use of PHAs is not currently realistic due to a variety of factors. One of the major constraints of large‐scale PHA production is the cost of carbon substrate for PHA‐producing microbes. The cost of production could potentially be reduced with the use of waste carbon from food‐related processes. Food wastage is a global issue and therefore harbours immense potential to create valuable bioproducts. This article's main focus is to examine the state of the art of converting food‐derived waste into carbon substrates for microbial metabolism and subsequent conversion into PHAs.

## Introduction

Microbially produced polyhydroxyalkanoates (PHAs) are among the most well‐studied biologically derived plastics. This is due to their suitability as potential replacements for petrochemically derived plastics because they are biodegradable and biocompatible (Bordes *et al*., 2009; Chanprateep, 2010). PHAs are carbon‐based polymers naturally created to store excess carbon sources and maintain energy balances (Escapa *et al*., 2012). Under certain conditions, such as nitrogen, phosphorus or oxygen limitation in the presence of excess carbon sources, some microorganisms accumulate high concentrations of PHAs (Anderson and Dawes, 1990; Lee, 1996).

There are over 155 confirmed unique PHA monomer subunits, which demonstrates the diversity of potential PHA polymers that can be produced using microorganisms (Agnew and Pfleger, 2013). The diversity of available monomers could lead to many different applications, as each resulting polymer has different material properties. For example, the melting temperatures of PHAs range from 50 to 180 °C and crystallinities of PHAs range from 30 to 70% (Rehm, 2010). Polyhydroxybutyrate (PHB), a short‐chain‐length (scl) PHA, is by far the most well‐studied PHA polymer and is able to accumulate to high concentrations in cells growing on a variety of carbon substrates (Reddy *et al*., 2003). For example, *Cupriavidus necator* has been recorded to have as high as 74% of its cell weight as PHB and recombinant *Escherichia coli* have been recorded to accumulate up to 85% of their dry cell weight as PHB (Kim *et al*., 1994; Wang *et al*., 2009). Some potential applications of PHAs could include commercial packaging (Bhardwaj *et al*., 2014; Bugnicourt *et al*., 2014), agricultural purposes (Akaraonye *et al*., 2010) and medical uses (Wu *et al*., 2009; Dinjaski and Prieto, 2015; Mozejko‐Ciesielska and Kiewisz, 2016). Although it is hoped that PHAs will be able to replace petrochemical plastics in these areas as production processes improve, the cost of production is currently prohibitive to all applications except higher‐value medical uses (Keshavarz and Roy, 2010; Chen and Patel, 2011; Zhu *et al*., 2016).

While the properties of PHAs seem suitable as potential petrochemical plastic replacements, there are still bottlenecks for scaling up microbial production systems. One of the major bottlenecks is the cost of carbon substrates, which have been estimated to be 28–50% of the total production process (Choi and Lee, 1999; Obruca *et al*., 2015; Strong *et al*., 2016). There are a number of complex waste streams that can potentially act as carbon substrates for microbial PHA manufacture, such as waste streams from biodiesel production (Kenny *et al*., 2012; Escapa *et al*., 2013), municipal wastewater (Rahman *et al*., 2014, 2015), agricultural waste (Linton *et al*., 2012), syngas production (Drzyzga *et al*., 2015), traditional plastic waste (Wierckx *et al*., 2015) and others (Gomez *et al*., 2012). Food waste is a prime candidate for an inexpensive carbon source, due to its wide spread availability and the potential to solve significant waste problems when used to produce PHAs.

Food wastage is a global problem and occurs at different stages in food production systems, starting from the harvesting of food to storage, packaging and end of life (Parfitt *et al*., 2010). In Europe, it is estimated that approximately 88 million tons of food is wasted and of this, 57 million tons is from households and food service (Stenmarck *et al*., 2016). In the United States, the Environmental Protection Agency (EPA) estimated that in 2013 approximately 37 million tons of food ended up in municipal solid waste systems, which was approximately 14% of all waste in the United States (US EPA, 2015). Another factor that is coupled to food waste is the energy that is lost in producing, processing and transporting the waste. This energy has been reported to be upwards of 2000 trillion British thermal units (BTU) in the United States, equivalent to 2.11 × 10^12^ megajoules (MJ) (Cuéllar and Webber, 2010). If an alternative means of transforming food waste into value‐added products are developed, then energy is essentially being transformed into useful products. Food waste conversion to tangible bioproducts has gathered plenty of attention, with systems being developed to produce a wide range of value‐added products such as biofuels, materials and a variety of additional feedstock chemicals (Lin *et al*., 2013; Pfaltzgraff *et al*., 2013; Matharu *et al*., 2016).

**Figure 1:**
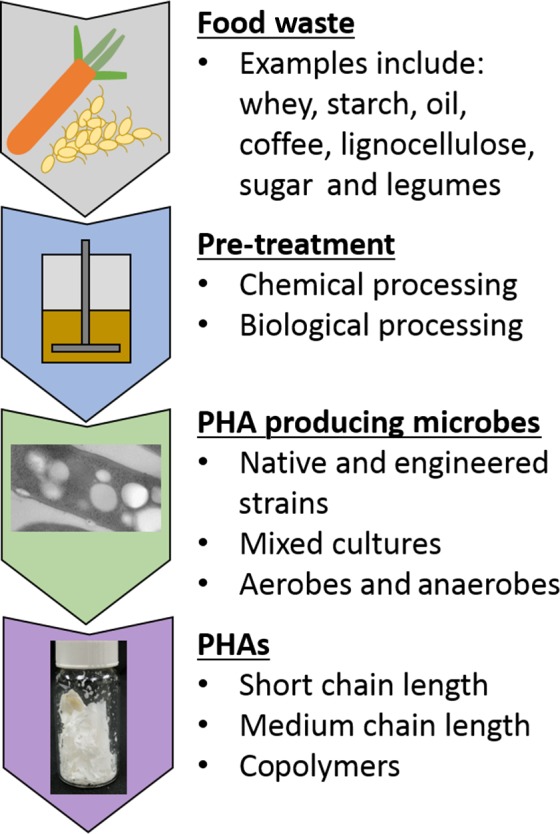
Conversion of food waste into PHAs using PHA‐producing microbes. Conversion of food waste often requires a pre‐treatment step where complex food waste is broken down into subcomponents. PHA‐producing microbes can then metabolize the carbon substrate and accumulate the biopolymer PHA.

The main objective of this review is to summarize the current state of PHA production from food waste using microbes, as depicted in Fig. 1. More specifically, the purpose of this investigation was to study systems that convert a variety of food wastes into microbially‐derived PHAs. Some considerations taken into account were as follows: food waste pre‐treatment steps, scalability, bioreactor design, microorganisms used and final PHA polymer produced.

## PHAs from food waste using pure cultures

There are many different systems that have been proposed to convert food waste into PHAs as there are numerous waste streams generated by food production, processing and use. Each food source has its own complexities and requires different pre‐treatments, bacterial strains, culturing conditions and downstream processing. Often, the organics associated with food wastes are complex compounds that cannot be directly used by PHA‐producing organisms (Anderson and Dawes, 1990). In these cases, a pre‐treatment or processing method is necessary to convert the complex molecules found in food waste into PHA precursors. Precursors include simple sugars like glucose or lactose and fatty acids like acetic or propionic acids. Many of the simpler food wastes are hydrolysed to convert the food waste into suitable precursor molecules and then fed directly to a pure culture of an appropriate microorganism. Whey, starch, oils, lignocellulosic materials, legume and sugar wastes each have methods proposed to create PHAs.

### PHAs from whey

One of the food wastes of interest is dairy whey. Whey is a by‐product of the cheese‐making process that consists of lactose, proteins, fats, water‐soluble vitamins, mineral salts and other essential nutrients for microbial growth. Although whey may be used as a source for producing lactose, casein and protein powder, it is estimated that approximately 50% of whey is still disposed of in wastewater treatment plants or used in animal feed (Pescuma *et al*., 2015). One study indicated that 1.15 × 10^8^ to 1.60 × 10^8^ tons of whey were produced worldwide, surpassing the requirements for whey powder production (Koller *et al*., 2013). When whey is used to produce proteins like lactoferrin or lactoferricin, lactose‐rich whey retentate remains as a waste material that must be disposed. Furthermore, acid whey is a by‐product of cottage cheese, cream cheese and Greek yogurt manufacturing. While acid whey can be used as animal feed, it is difficult to process into traditional whey protein concentrates due to the high acid content (Chandrapala *et al*., 2015; Lievore *et al*., 2015; Pescuma *et al*., 2015; Ryan and Walsh, 2016). Disposal of whey is currently a notable problem facing the dairy industry, making it a potentially inexpensive carbon source for PHA production (Girotto *et al*., 2015).

In addition to being a low‐cost carbon source, whey has the advantage of not requiring extensive pre‐treatment for use in fermentation via hydrolysis using enzymes or acid methods (Gomez *et al*., 2012). A life cycle assessment demonstrated that optimized production of PHAs from whey has a comparable ecological footprint to that of producing petroleum‐based plastics and is superior to producing whey powder (Koller *et al*., 2013). This life cycle assessment was based on producing PHAs using the archaeal production strain *Haloferax mediterranei* DSM 1411. Producing whey powder was inferior because it was a low market value product that used high amounts of energy to concentrate whey by evaporation. The main limitations on PHA production were found to be energy requirement for the fermentation process and a low amount of PHA output per kg whey input.

Most studies that used whey to produce PHAs have involved recombinant *Escherichia coli*, which will be described in a subsequent section. *E. coli* was selected because many of the traditional PHA‐producing microorganisms cannot directly metabolize whey. Studies have also examined using organisms such as the halophile archaeon *H. mediterranei* (Koller, 2015; Pais *et al*., 2016), an unidentified highly osmophilic organism (Koller *et al*., 2005); *Thermus thermophiles* HB8 (Pantazaki *et al*., 2009); and *Cupriavidus necator* DSM 545 transformed to include the *lacZ*,* lacI* and *lacO* genes of *E. coli* (becoming *C. necator* mRePT) (Povolo *et al*., 2010) (see Table 1). Particularly notable is the fact that *H. mediterranei* produces the copolymer poly‐(3‐hydroxybutyrate‐co‐3‐hydroxyvalerate) (PHBV), while *T. thermophiles* HB8 produced a unique combination of 3‐hydroxyvalerate (3HV) with medium‐chain‐length (mcl) PHAs. Both of these polymer blends have improved ductility compared to PHB, making them preferable for industrial use (Reddy *et al*., 2003; Rehm, 2010). Apart from *C. necator* mRePT, these organisms are capable of utilizing whey to produce PHAs with more desirable properties than PHB.

**Table 1:** Production of PHAs from food waste using pure microbial cultures

Food waste source	Microorganisms(s)	PHA polymer type	Cultivation	Dry cell weight (g l^−1^)	Maximum PHA production reported (g PHA g^−1^ dcw)	References
Whey	Highly osmophilic organism	PHBV	Fermenter, batch	NA	8–10%	Koller *et al*. (2005)
Whey	*Haloferax mediterranei*	PHBV	Fermenter, fed‐batch	10.91	66%	Koller (2015)
Whey	*Haloferax mediterranei*	PHBV	Fermenter, batch	7.45	53%	Pais *et al*. (2016)
Whey	*Thermus thermophiles* HB8	PHV and mcl‐PHAs	Flask, batch	1.60	35.6%	Pantazaki *et al*. (2009)
Whey permeate	*Cupriavidus necator* mRePT	PHB	Flask, batch	8	25%	Povolo *et al*. (2010)
Cassava starch wastewater	*Cupriavidus* sp. KKU38	PHB	Flask, batch	9.69	61.60%	Poomipuk *et al*. (2014)
Starch	*Azotobacter chroococcum*	PHB	Fermenter, batch	54	46%	Kim (2000)
Soy bean and rapeseed oil	*Cupriavidus necator* H16	PHB	Fermenter, two‐stage batch	6.1	57%	Taniguchi *et al*. (2003)
Soy bean, rapeseed and corn oil and lard	*Cupriavidus necator* H16	PHB	Fermenter, two‐stage batch	6.5	79%	Taniguchi *et al*. (2003)
Palm oil and lard	*Cupriavidus necator* H16	PHB	Fermenter, two‐stage batch	6.8	83%	Taniguchi *et al*. (2003)
Tallow	*Cupriavidus necator* H16	PHBV	Fermenter, two‐stage batch	5.8	80%	Taniguchi *et al*. (2003)
Waste frying rapeseed oil	*Cupriavidus necator* H16	PHB	Flask, batch	10.8	67.9%	Obruca *et al*. (2014b)
Waste frying palm oil	*Cupriavidus necator* H16	PHB	Flask, batch	11.9	58.0%	Obruca *et al*. (2014b)
Waste frying sunflower oil	*Cupriavidus necator* H16	PHB	Flask, batch	10.812.53	52.4%	Obruca *et al*. (2014b)
Corn oil	*Psuedomonas* species	mcl‐PHA	Flask, batch	12.53	35.63%	Chaudhry *et al*. (2011 *)*
Spent coffee grounds oil	*Cupriavidus necator* DSM 428	PHB	Fermenter, fed‐batch	16.7	78.40%	Cruz *et al*. (2014)
Spent coffee Grounds oil	*Cupriavidus necator* H16	PHB	Fermenter, fed‐batch	55.4	89.10%	Obruca *et al*. (2014b)
Spent coffee grounds hydrolysate	*Burkholderia cepacia*	PHBV	Flask, batch	4.91	54.79%	Obruca *et al*. (2014a)
Oil palm empty fruit bunch	*Bacillus megaterium* R11	PHB	Flask, batch	24.29	51.60%	Zhang *et al*. (2013)
Wheat straw	*Burkholderia sacchari* DSM 17165	PHB PHB	Fermenter, fed‐batch	146	72%	Cesário *et al*. (2014)
Rice straw	*Bacillus firmus* NII 0830	PHB	Fermenter, batch	1.9	89%	Sindhu *et al*. (2013)
Wheat bran	*Halomonas boliviensis* LC1	PHB	Fermenter, batch	1.08	34%	Van‐Thuoc *et al*. (2008)
Tequila bagasse	*Saccharophagus degradans*	PHA	NA	NA	> 0%	Munoz and Riley (2008)
Molasses	*Psuedomonas* species	PHA	Flask, batch	10.54	20.63%	Chaudhry *et al*. (2011)
Fermented mash	*Psuedomonas* species	PHA	Flask, batch	7.02	23.56%	Chaudhry *et al*. (2011)
Spent wash	*Psuedomonas* species	PHA	Flask, batch	8.56	25.46%	Chaudhry *et al*. (2011)
Sugarcane molasses	*Bacillus megaterium* BA019	PHB	Fermenter, fed‐batch	72.2	42%	Kulpreecha *et al*. (2009)
Sugar beet juice	*Alcaligenus latus*	PHB	Flask, two‐ stage batch	4.01	38.66%	Wang *et al*. (2013)
Sugarcane bagasse	*Burkholderia* sp.	PHB	Fermenter, fed‐batch	6.8	48%	Lopes *et al*. (2014)
Sugarcane bagasse	*Cupriavidus necator*	PHB	Flask, batch	NA	57%	Yu and Stahl (2008)
Sugarcane vinasse	*Haloarcula marismortui*	PHB	Flask, batch	12	23%	Pramanik *et al*. (2012)
Sugarcane vinasse	*Haloferax mediterrranei*	PHBV	Flask, batch	28.1	70%	Bhattacharyya *et al*. (2012)
Rice‐based ethanol stillage	*Haloferax mediterrranei*	PHBV	Flask, batch	23	71%	Bhattacharyya *et al*. (2014)
Malt waste	*Alcaligenus* e*utrophus* DSM1124	PHB	Fermenter, batch	32.36	70%	Yu *et al*. (1998)
Soy waste	*Alcaligenus eutrophus* DSM1124	PHB	Fermenter, batch	18.42	32.57%	Yu *et al*. (1999)
Bean curd waste	*Alcaligenus latus*	PHB	Flask, batch	3.73	66.56%	Kumalaningsih *et al*. (2011)

NA is not available.

### PHAs from starch

Starch is another carbon source derived from food wastes that has been studied for production of PHAs. Starch is a glucose polymer produced by plants such as rice, wheat, potatoes, maize and cassava. Although starch is readily consumed by humans, there are some significant starchy waste streams from food production that can be used by PHA‐producing microorganisms. In the work of Poomipuk *et al*. (2014), *Cupriavidus* sp. KKU38 isolated from cassava starch wastewater was used to produce PHAs from cassava starch hydrolysate. This study demonstrated that under optimal conditions and nitrogen starvation, *Cupriavidus sp*. could produce a moderately high biomass concentration of 5.97 g l^−1^, with a PHA content of 61.6% (Table 1). A study by Kim (2000) avoided using expensive enzymes to hydrolyse starch using *Azotobacter croococcum*, a type of bacteria that can digest starch directly. In this study, 54 g l^−1^ dry cell weight with 46% PHB was obtained with oxygen limitation (Table 1). These studies indicate that high concentrations of cells containing PHAs are possible on starchy food wastes, even with minimal pre‐treatments in the case of the Kim (2000) study.

### PHAs from waste oil

Waste oils from both household and industrial applications are potential carbon sources for producing PHAs. These oils generally require no pre‐treatment and may be added directly to media as a carbon substrate. A study by Taniguchi *et al*. (2003) investigated the use of *Cupriavidus necator* H16 to convert waste oils and tallow to PHAs. The highest amount of PHA produced in this study came from using palm oil and lard as carbon sources, achieving a dry cell weight of 6.8 g l^−1^ and a PHB accumulation of 83%. Another notable finding is that when tallow was used as a carbon source, the copolymer PHBV was produced instead of pure PHB (Table 1).

Obruca *et al*. (2014b) also tested waste frying rapeseed oil, waste frying palm oil and waste frying sunflower oil as carbon sources for PHA production via *C. necator* H16. They demonstrated dry cell weights (g l^−1^) and PHB contents (%) of 10.8 and 67.9%, 11.9 and 58.0%, and 10.8 and 52.4%, respectively, for the different oil types. Chaudhry *et al*. (2011) used corn oil and found that a *Pseudomonas* strain could achieve a dry cell weight of 12.53 g l^−1^ with a mcl‐PHA content of 35.63% (Table 1). Although lower quantities of PHA were produced by the *Pseudomonas* species used by the Chaudhry group when compared to the *C. necator* H16 used by the Obruca group, the mcl‐PHA produced by the *Pseudomonas* strain when it was fed corn oil is more desirable than PHB. These studies indicate that using waste oils as a carbon source allow cells to produce high concentrations of PHAs relative to dry cell weight in low titres of cells.

### PHAs from spent coffee grounds

An alternative oil waste from food is spent coffee grounds (SCG) oil. SCG are produced during coffee processing and consumption. Approximately 9–15% of the grounds is oil that can be extracted for use (Al‐Hamamre *et al*., 2012). The remaining portion of the spent coffee grounds is primarily lignocellulosic materials that can be combusted for heat or hydrolysed and converted into PHAs by *Burkholderia cepacia* (Obruca *et al*., 2014a). Obruca *et al*. (2014b) directly compared use of SCG oil to other waste oils in *C. necator* H16 and found that the SCG oil was superior for PHB production. In a shake flask experiment, the SCG oil produced a dry cell weight of 14.2 g l^−1^ with a PHB content of 70.3% compared to the values for the waste oils discussed previously (Table 1). When scaled up, the SCG oil achieved an impressive dry cell weight of 55 g l^−1^ with a PHB concentration of 89.1% in fed‐batch mode (Table 1). The main difficulty encountered is that the SCG oil is a natural foaming agent; however, other plant oils, such as waste frying oils, can be added to serve as both carbon sources and as antifoaming agents.

One study by Cruz *et al*. (2014) used supercritical fluid extraction with CO_2_ (scCO_2_) to extract the SCG oil and then fed it directly to *C. necator* DSM 428 in fed‐batch mode. The culture reached a dry cell weight of 10.7 g l^−1^ with a PHB content of 78.4% (Table 1). In the same study, batch mode operation was also used to produce PHB from SCG oil, but this approach produced lower amounts of PHB in comparison with the fed‐batch mode. The maximum biomass accumulation observed in batch mode was up to 55% (w/w) of PHB, which yielded a polymer concentration of 6 g l^−1^, in comparison with a polymer concentration of 13.1 g l^−1^ observed in the fed‐batch mode. The main difference between the study by the Cruz group and the study by the Obruca group was that the Cruz group used an extraction method that avoided the use of hazardous organic solvents like *n*‐hexane. As such, the Cruz process may be superior for mass production processes despite achieving lower dry cell weights. Both studies indicate that SCG oils are a carbon source with great potential for PHA production.

### Lignocellulosic waste conversion to PHAs

Lignocellulosic materials are tough plant‐based materials that are made of cellulose, pectin, hemicellulose and lignin. Examples of this type of waste from food industry include bagasse, rice straw, wheat straw and bran. Waste streams of lignocellulosic compounds generally require hydrolysis to convert them into fermentable sugars and then detoxification to remove inhibitory compounds produced during hydrolysis, as reviewed in Obruca *et al*. (2015). A variety of lignocellulosic materials have been investigated for PHA production, including oil palm empty fruit bunch (Zhang *et al*., 2013), wheat and rice straw (Sindhu *et al*., 2013; Cesário *et al*., 2014), wheat bran (Van‐Thuoc *et al*., 2008), sugarcane bagasse (Yu and Stahl, 2008) and tequila bagasse (Munoz and Riley, 2008) (see Table 1). Despite pre‐treatments, the lignocellulosic materials often resulted in low levels of cell growth. One of the more promising lignocellulosic processes, however, was investigated by Cesário *et al*. (2014). In this study, an ammonia fibre expansion (AFEX) process was used as pre‐treatment followed by an enzymatic hydrolysis of the cellulose and hemicellulose fractions of ground wheat straw to produce glucose, xylose and arabinose. The hydrolysate was fed to *Burkholderia sacchari* DSM 17165 in a fed‐batch fermentation process. A biomass concentration of 146 g l^−1^ with a PHA concentration of 72% was achieved using this method (Table 1). While lignocellulosic materials generally require extensive pre‐treatment, they do offer some potential as carbon substrates.

### PHAs from sugar industry waste

Several waste streams from the sugar industry have been investigated for their PHA‐producing potential. One example is low‐grade molasses, which is a residual syrup generated in sugar‐refining mills that is high in sucrose, but not suitable for food (Gomez *et al*., 2012). Most studies using molasses indicate that cell production and polymer content are not currently cost competitive. For example, Chaudhry *et al*. (2011) used a *Pseudomonas* species to convert sugar industry wastes to PHAs, and found that the dry cell weight and PHA contents were 7.02–12.53 g l^−1^ and 20.63–35.63%, respectively, with molasses functioning the best overall (Table 1). One promising study by Kulpreecha *et al*. (2009) used sugarcane molasses as the main carbon source for *Bacillus megaterium* BA‐019 to achieve a dry cell weight of 72.7 g l^−1^ in 24 h, with a PHB content of 42%. This latter study does indicate that molasses may be used to produce a considerable amount of PHB.

Sugar beet is another industrial waste with high sucrose content. *Alcaligenes latus* (ATCC 29714) was demonstrated to grow in sugar beet juice with supplemental nutrients to achieve optimal growth of 10.3 g l^−1^ and a PHB content 38.66% (Table 1) (Wang *et al*., 2013). The Italian company, Bio‐on, has also developed a range of PHA polymers using local sugar beet juice (Dietrich *et al*., 2017). The company now uses sugar beet and sugar cane wastes from around the world to produce PHAs for use in cosmetics and pharmaceuticals (Bio‐on, 2017).

Bagasse, the lignocellulosic residue of crushed sugar beets or sugar cane stalks, has been examined as a source of xylose for PHA production (Silva *et al*., 2014; Sindhu *et al*., 2016). Bagasse requires a pre‐treatment step to convert it to digestible sugars and to remove inhibitory compounds like formic acid, acetic acid and furfural. In a study by Lopes *et al*. (2014), for example, acid‐treated sugarcane bagasse at 120 °C produced 3.264 g l^−1^ PHB in *Burkholderia* sp. In addition, PHBV was produced when levulinic acid was added. Yu and Stahl also used acid and moderate heat (100–130 °C) to pre‐treat sugarcane bagasse for use as a carbon source for *C. necator*. The inhibitory effects of the solution were overcome by a large inoculum of a tolerant strain of *C. necator* and a diluted hydrolysate solution which yielded PHB to 57% dry cell weight (Yu and Stahl, 2008). This demonstrates that bagasse can not only be used as a fuel for boilers and as a raw material for paper, but also as a carbon source for PHA production.

Another significant sugar industry waste is vinasse, an acidic compost with a pH of 3.5–5.0 and high organic content. Recent research into using vinasse as a carbon source for PHA production has focused on using extremely halophilic archaea like *Haloarcula marismortui* and *H. mediterranei* (Bhattacharyya *et al*., 2012; Pramanik *et al*., 2012). These organisms have the advantage of not requiring a sterile environment due to the high salinity of fermentation broth and are also notable for being able to produce PHBV without the addition of organic acids as precursors. One of the main drawbacks of using halophilic organisms is disposing the saline solution after fermentation, which Bhattacharyya *et al*. (2014) addressed using a two‐stage desalination of spent stillage medium to reuse medium salts, tested with rice‐based ethanol stillage as a carbon source. In addition, vinasse contains polyphenolic inhibitory compounds that make pre‐treatment such as adsorption on activated carbon necessary to use vinasse as a carbon source in concentrations above 10% (Bhattacharyya *et al*., 2012; Pramanik *et al*., 2012). After pre‐treating vinasse, concentrations of up to 50% were used with *H. mediterranei* to produce 17.4–19.7 g l^−1^ PHA (Bhattacharyya *et al*., 2012) and 100% vinasse was used with *H. marismortui* to generate 4.5 ± 0.2 g l^−1^ PHA (Pramanik *et al*., 2012).

### PHAs from legume waste

Legumes have also been demonstrated as suitable carbon sources for PHA production. In a study by Kumalaningsih *et al*. (2011), liquid bean curd waste supplemented with an initial sucrose concentration of 25 g l^−1^ was fed to *A. latus*. A dry cell weight of 3.73 g l^−1^ with a PHA content of 66.56% was observed after 60 h of culturing (Table 1). Soya waste was used by Yu *et al*. (1998, 1999) to produce PHAs using *Alcaligenus eutrophus* DSM 1124, but it was found that the organism was more successful at converting malt waste to PHAs than soy, with 32.57% PHA accumulated out of 18.42 g l^−1^ dcw for soy compared to 70% PHA out of 32.36 g l^−1^ dcw for malt (Table 1).

Pure cultures grown on food wastes tend to promote high cell growth and accumulation of PHAs. Some substrates, such as starch or SCG oil, are more promising than others, such as molasses or waste oils. While using waste food substrates to feed these cultures reduces costs due to carbon source, pre‐treatments are often necessary. Most of the traditional bacteria used for PHA production produce the PHB polymer, and the cost of sterilization and oxygen supply for cultures are not economically favorable. Scale‐up and optimization of processes may be able improve these difficulties. Using processes that require less energy expenditure may also reduce costs. For example, using the halophilic organism *H. mediterranei* has the dual benefits of making sterilization unnecessary and producing PHBV polymer instead of PHB alone. Even with these drawbacks, using pure cultures to produce PHAs from food wastes has promise.

## Recombinant microbes for PHA production

Natively accumulating microbial strains are most commonly utilized for conversion of food‐based carbon substrates into PHAs. The use of recombinant organisms, however, could be advantageous as the microorganisms can be triggered to produce PHAs without inducing stressed conditions, such as nitrogen or phosphorus starvation, which could potentially lead to cost savings. In addition, recombinant microorganisms are well defined and thus could be further engineered for optimization. Bacteria known to be able to utilize certain substrates that the native PHA producers cannot use may also be transformed with PHA‐producing genes to produce PHA from food wastes like whey, starch or oils. Furthermore, culturing recombinant microbes could allow faster growth and turnaround times for bioreactors.

*Escherichia coli* is the standard organism used in genetic engineering and has been shown to be advantageous for producing PHAs. Some strains of *E. coli* are known to be able to utilize lactose, a substrate that many PHA‐producing organisms like *C. necator* are not able to metabolize. As such, most studies using high lactose containing dairy whey as a carbon source have used *E. coli* with the PHA‐producing genes (the *pha* operon) from *C. necator*. Traditional laboratory strains of *E. coli* like XL1‐Blue, JM or DH5α often lack the ability to utilize lactose as a nutrient source, which has made developing alternative strains from wild‐type *E. coli* cells necessary. When nine different strains derived from wild‐type cells were tested for their ability to produce PHA using lactose as a sole carbon source, it was documented that strains GCSC4401 and GCSC6576 transformed with a high‐copy‐number plasmid, pSYL107 containing the *A. eutrophus* PHA biosynthesis operon, were best able to produce PHAs. The maximum PHB concentration and PHB content obtained were 5.2 g l^−1^ and 81% of dry cell weight respectively (Table 2) (Lee *et al*., 1997).

**Table 2:** Production of PHAs from food waste using recombinant bacteria

Food waste source	Strain	Plasmid	PHA operon origin	PHA polymer type	Cultivation	Dry cell Weight (g l^−1^)	Maximum PHA Production Reported (g PHA g^−1^ dcw)	References
Whey	*E. coli* GCSC657	pSYL107	*C. necator*	PHB	Shake flask	6.4	81%	Lee *et al*. (1997)
Whey	*E. coli* GCSC657	pSYL107	*C. necator*	PHB	Fermenter, fed‐batch	87	80%	Wong (1998)
Whey	*E. coli*	pSYL107	*C. necator*	PHB	Fermenter, fed‐batch	31	80%	Kim (2000)
Whey	*E. coli* CML3‐1	pMAB26	*C. necator*	PHB	Fermenter, fed‐batch	33.09	28.65%	Pais *et al*. (2014)
Whey	*E. coli* CGSC 4401	pJC4	*Alcaligenes latus*	PHB	Fermenter, fed‐batch	119.5	80.50%	Ahn *et al*. (2000)
Whey	*E. coli* CGSC 4401	pJC4	*Alcaligenes latus*	PHB	Fermenter, fed‐batch	194	87%	Ahn *et al*. (2001)
Whey	*E. coli*	pJC4	*Alcaligenes latus*	PHB	Fermenter, fed‐batch	14.5	71%	Park *et al*. (2002)
Whey	*E. coli* K24K	pJP24K	*Azotobacter* sp. FA8	PHB	Fermenter, fed‐batch	70.1	72.90%	Nikel *et al*. (2006)
Malt waste	*E. coli*	pUC19/PHA	*C. necator*	PHBV	Fermenter, fed‐batch	NA	16%	Law *et al*. (2004)
Soy waste	*E. coli*	pUC19/PHA	*C. necator*	PHBV	Fermenter, fed‐batch	NA	23%	Law *et al*. (2004)
Soy waste	*E. coli* XL1‐Blue	pKS	*C. necator*	PHB	Fermenter, batch	3.025	27.83%	Hong *et al*. (2000)
Organic acids	*E. coli* pnDTM2	NA	NA	PHB	Fermenter, batch	2.9	45%	Eshtaya *et al*. (2013)
Starch	*Aeromonas* Sp. KC007‐R1	pRK415 H16	*C. necator*	PHB	Fermenter, batch	1.83	32.70%	Chien and Ho (2008)

NA is not available.

Initial studies using recombinant *E. coli* showed potential, and subsequent studies focused on improving production. The same group that developed *E. coli* strains GCSC4401 and GCSC6576 published another study focusing on scale‐up. *E. coli* GCSC6576 (pSYL107) was grown on a high concentration of whey in a fed‐batch system, where a dry cell weight of 87 g l^−1^ and PHB content of 79% was achieved (Table 2) (Wong, 1998). A follow‐up study improved on these methods by controlling the timing of PHB biosynthesis in recombinant *E. coli* using lactose concentrations. This allowed *E. coli* strain GCSC6576 (pSYL107) to accumulate PHA concentrations up to 80% of the dry cell weight without removing culture broth (Table 2) (Kim, 2000). As such, *E. coli* has been successfully used to produce PHAs from whey using the *pha* operon from *C. necator*.

Other studies have also used *E. coli* to produce PHAs on whey using a PHB operon from *A. latus* rather than *C. necator*. Several strains of *E. coli* that were known to be able to utilize lactose were transformed with plasmid pJC4 with *A. latus pha* genes. It was found that strain CGSC4401 was the ideal strain and dry cell weight and PHB content of 119.5 g l^−1^ and 80.5% were achieved (Table 2) (Ahn *et al*., 2000). This study and other early studies had problems with volumetric limitations of fermenter due to the low solubility of lactose in water and low PHB productivity. The former was partially addressed by highly concentrated whey solution. A subsequent study by Ahn *et al*. (2001) used a cell recycle membrane fed‐batch system to increase PHB productivity, achieving a final cell concentration and PHB content of 194 g l^−1^ and 87% respectively (Table 2). In a third study, the same recombinant strain grown in whey in a 30‐l fermenter (26 h) and 300‐l fermenter (20 h) produced 70% and 67% PHB, respectively, demonstrating the process of using whey with this strain was scalable. (Park *et al*., 2002).

Two other studies used recombinant *E. coli* to produce PHAs from whey. Pais *et al*. used proton suicide methodology to select for a recombinant strain of *E. coli* that synthesized a low amount of organic acids after it was transformed with the *C. necator pha* operon. The results indicated that the lower organic acid production resulted in slower growth, but a higher production of PHB (18.88 g PHB l^−1^ versus 7.8 g PHB l^−1^ in the original transformed strain) (Pais *et al*., 2014). Another study used recombinant *E. coli* harbouring the PHB biosynthetic genes from *Azotobacter* sp. strain FA8 to produce PHB from whey and corn steep liquor as the main carbon and nitrogen sources. The maximum cell density and PHB concentrations attained were 70.1 g l^−1^ and 73% (Table 2) (Nikel *et al*., 2006). These studies demonstrate the use of metabolic engineering to improve production of PHA and another option for PHA‐producing genes that can be used in *E. coli*.

While whey is the primary food waste substrate investigated for recombinant *E. coli*, other nutrient sources have been pursued. A study by Hong *et al*. (2000) successfully cloned the *pha* operon from *C. necator* into *E. coli* XL1‐Blue and demonstrated PHB production from soya waste. Soya waste was hydrolysed in NaOH for 8 h and then fed into a batch 3‐l fermenter, and 27.83% PHB accumulation was observed after 9 h of culturing (Table 2). The same group later demonstrated recombinant production of PHBV using *E. coli* HMS174 with a plasmid containing the *pha* operon. Malt and soya waste were obtained locally and recombinant *E. coli* accumulated up to 16% and 23% PHAs, respectively (Table 2). As a comparison, when this strain was grown in glucose, it produced approximately 43% dry cell weight PHAs (Law *et al*., 2004). This demonstrates that soya and malt waste are potential carbon sources for PHA production.

In addition to malt and soya waste, another study used locally procured restaurant waste that was anaerobically digested to produce lactic and acetic acids. Similar to processes mentioned previously, these precursors were fed to recombinant *E. coli* pnDTM2, which accumulated 44% PHB (Table 2) (Eshtaya *et al*., 2013). While using restaurant waste required an extra step to convert the complex mixture into organic acids, it provided an opportunity to use an inexpensive and widely available carbon source.

Many studies that use recombinant bacteria to produce PHAs from food waste focus on using *E. coli*, which is the common workhorse of molecular biology. In addition to *E. coli*, another *Aeromonas* sp. (strain KC007‐1) has also been used. The strain was chosen for its ability to directly use starch as a carbon source, and the *pha* operon from *C. necator* H16 was added to increase production rates of PHAs. In this case, the bacteria were able to accumulate 32.7% PHA (Chien and Ho, 2008), indicating that *E. coli* is not the only organism to be successfully modified to produce PHAs from food waste.

The examples mentioned here showed that non‐native microorganisms were able to successfully produce high amounts of PHAs. In all cases, pathways for PHA production were transferred from a native host to the non‐native microorganism. As genetic tools increase, it will be possible to optimize pathways for non‐native hosts to consume substrates and produce PHAs. The idea to genetically engineer microbial strains with a dual purpose of consuming inexpensive substrates and producing valuable bioproducts has been mentioned previously (Gustavsson and Lee, 2016), although relatively few studies in the past 10 years have used recombinant bacteria to produce PHAs from food waste. Future research into scale‐up and maintaining high productivity, cell concentrations and PHA content is still necessary to make PHA production from food wastes using recombinant bacteria feasible on an industrial scale.

## Production of PHAs from food waste using anaerobic digestion

One method that has been used to biologically convert complex food wastes to PHA precursors is anaerobic digestion using open systems of mixed microbial cultures (MMCs). The general process that has been followed in most studies using food waste has involved three steps: (i) acidogenic fermentation; (ii) culture selection; and (iii) PHA accumulation. The primary advantage to this approach is that MMCs have lower investment and operating costs, because substrate pre‐treatment processes are not required, sterilization is typically not necessary and less expensive carbon sources can be used (Reis *et al*., 2003; Gurieff and Lant, 2007). This is important, as analyses have indicated that the high energy expenditure used in sterilization, aeration and agitation to produce PHAs in pure cultures causes these bioplastics to have little advantage over traditional synthetic plastics in environmental impact (Gomez *et al*., 2012; Koller *et al*., 2013). In addition, due to the greater variety of organisms working together with complex substrates in MMCs, more diverse PHAs, such as polyhydroxyhexanoate (PHH), polyhydroxyoctanoate (PHO) and polyhydroxydecanoate (PHD), are produced (Chae and Shin, 2007). This indicates that MMCs are a potentially viable option for producing PHAs from food waste.

The first step in producing PHAs from MMCs is acidogenic fermentation. During this step, complex wastes, such as food scraps, spentwash and wastewater, are broken down into simpler and smaller fermentative acids, mainly C2‐C4 acids such as acetic, propionic, butyric and lactic acids (Serafim *et al*., 2008; Albuquerque *et al*., 2012). A variety of feedstocks have been used for anaerobic production of volatile fatty acids (VFAs) from food waste, including food scraps from restaurants or kitchens (Zhang *et al*., 2007; Hafuka *et al*., 2011; Omar *et al*., 2011; Eshtaya *et al*., 2013), whey (Duque *et al*., 2014; Valentino *et al*., 2015; Colombo *et al*., 2016), jowar grain spentwash and rice spentwash (Khardenavis *et al*., 2007), tomato cannery wastewater (Liu *et al*., 2008), olive oil mill pomace and wastewater (Waller *et al*., 2012; Campanari *et al*., 2017), palm oil mill effluent (Din *et al*., 2006; Salmiati *et al*., 2007), sugarcane molasses (Albuquerque *et al*., 2007, 2011, 2012), pea shell waste (Patel *et al*., 2012), condensate of food waste (Chae and Shin, 2007) and fermented brewery wastewater (Mato *et al*., 2008; Ben *et al*., 2016) (see Table 3 and Table 4). Applying pre‐treatments such as filtering and deproteinization to these waste streams has been examined, with mixed results (Khardenavis *et al*., 2007; Liu *et al*., 2008). Another pre‐treatment is buffering the waste solution to keep pH between 5.5 and 7.0, which has been shown to improve VFA production (Zeng *et al*., 2006; Waller *et al*., 2012; Eshtaya *et al*., 2013). A variety of food waste sources have been used to produce VFAs for use by cultures of bacteria that accumulate PHAs.

**Table 3:** Production of PHAs from anaerobically digested food waste

Food waste source	Microorganisms	PHA polymer type	Cultivation	Maximum PHA Production Reported (g PHA g^−1^ dcw)	References
Whey	Wastewater microbes	PHBV	Flask, batch	NA	Valentino *et al*. (2015)
Whey	Pre‐selected mixed microbial culture	PHBV	Flask, batch	81%	Colombo *et al*. (2016)
Whey	Activated sludge consortium	PHBV	Three‐stage reactors system	65%	Duque *et al*. (2014)
Sugarcane molasses	Activated sludge consortium	PHBV	Three‐stage reactors system	56%	Duque *et al*. (2014)
Brewery wastewater	Activated sludge consortium	PHBV	SBR	39%	Ben *et al*. (2016)
Food processing wastewater effluent	Activated sludge consortium	PHB	Flask, batch	60.70%	Khardenavis *et al*. (2007)
Jowar grain‐based distillery spentwash	Activated sludge consortium	PHB	Flask, batch	42.30%	Khardenavis *et al*. (2007)
Rice grain‐based distillery spentwash	Activated sludge consortium	PHB	Flask, batch	40%	Khardenavis *et al*. (2007)
Condensate of food waste	Enriched activated sludge consortium	PHBV and mcl‐PHAs	VSMBR	1.8%	Chae and Shin (2007)
Olive oil mill pomace	Activated sludge consortia	PHBV	SBR	39%	Waller *et al*. (2012)
Olive oil mill wastewater	Wastewater microbes	PHBV	SBR	11.3%	Campanari *et al*. (2017)
Tomato wastewater	Activated sludge consortium	PHA	Fermenter, batch	20%	Liu *et al*. (2008)
Fermented food waste	Wastewater microbes	PHA	Fermenter, anaerobic/aerobic	51%	Rhu *et al*. (2003)
Fermented molasses	Mixed microbial culture	PHBV	Fermenter, pulse feed	56%	Albuquerque *et al*. (2011)
Fermented molasses	Mixed microbial culture	PHBV	Fermenter, batch	60.50%	Albuquerque *et al*. (2012)

VSMBR is a vertical submerged membrane bioreactor; SBR is stirred batch reactor.

**Table 4:** Production of PHAs from organic acids derived from anaerobically digested food waste

Food waste source for organic acids	Microorganism	PHA polymer type	Cultivation	Dry cell Weight (g l^−1^)	Maximum PHA production Reported (g PHA g^−1^ dcw)	References
Restaurant waste	Recombinant *E. coli* pnDTM2	PHB	Fermenter, batch	2.9	45%	Eshtaya *et al*. (2013)
Restaurant waste	*C. necator* H16	PHB	Fermenter, continuous feeding	1.4	87%	Hafuka *et al*. (2011)
Food scraps from cafeteria	*C. necator*	PHBV	Fermenter, batch	22.7	72.60%	Du and Yu (2002)
Kitchen waste	*C. necator* CCGUG 52238	PHB	Fermenter, batch	4.6	52.79%	Omar *et al*. (2011)
Pea shells	*Bacillus cereus* Strain EGU3	PHB	Fermenter, batch	1.32	71%	Patel *et al*. (2012)

The second step of producing PHAs from mixed cultures is culture selection. During this step, bacteria are subjected to alternating conditions to obtain a microbial community where almost all microorganisms have a high PHA‐storing capacity and production rate. This is often carried out in sequencing batch reactors (SBRs), compact systems where the full feast and famine cycle is performed in one single reactor, and the length of each phase could be varied. The cycle may be either alternating conditions of external substrate excess (feast) and limitation (famine) in aerobic conditions or alternating anoxic and aerobic microenvironments. In both cases, limiting cell growth (through famine or anoxic conditions) increases PHA production and presents pressures that allow PHA‐producing strains to become predominant (Serafim *et al*., 2008). Using microautoradiography and fluorescence in situ hybridization (FISH), Albuquerque *et al*. (2012) found that in a culture grown on molasses, bacteria that their process selected for were primarily from the genera *Azoarcus, Thauera* and *Paracoccus*. Each of these populations specialized in digesting specific products of the acidogenic fermentation. *Azoarcus* and *Thauera* primarily consumed acetate and butyrate, respectively, while *Paracoccus* consumed a broader range of substrates. Other studies on culture selection have indicated that PHA‐storing bacteria in MMC are predominantly from the classes of *Alphaproteobacteria, Betaproteobacteria* and *Gammaproteobacteria*, as has been reviewed elsewhere (Serafim *et al*., 2008; Morgan‐Sagastume, 2016).

The third step of producing PHAs from MMCs is PHA accumulation. This step is designed to maximize PHA production in cells harvested from the enrichment bioreactor. The feast–famine cycle (FF) is known to increase synthesis and storage of PHA granules (Villano *et al*., 2010). Alternating anoxic and aerobic conditions have also been used in SBRs to improve PHA production from food waste. It has been documented that anoxic microenvironments tend to promote higher PHA accumulation due to better access to VFAs and lack of an electron acceptor, while aerobic environments tend to promote PHA degradation, but better nutrient removal (Venkateswar Reddy and Venkata Mohan, 2012). The alternating conditions pressure the cells to uptake nutrients and convert them to PHAs.

Several problems exist with MMCs. First, they generally have lower performance than pure cultures (when measured by volumetric productivity). This is due, in part, to lower cell concentrations that are usually found in MMCs. Often, concentrations are < 10 g l^−1^ compared to values greater than 100 g l^−1^ that can be found in pure culture studies. Further, information on the quality of PHAs produced in MMCs is scarce (Albuquerque *et al*., 2011). Another concern is that many of the organic acids produced during MMC fermentation inhibit bacterial growth, causing decreased productivity. These issues must be overcome to make MMCs a viable approach to producing PHAs from inexpensive carbon sources.

### Indirect coupling of MMC to PHA production

One approach to overcoming some of the difficulties associated with MMCs is using an indirect coupling approach where complex food wastes are digested in an MMC to produce VFAs, which are then harvested and fed to a pure culture fermentation (see Table 4). This increases the chance that PHA polymers will be consistent in their quality and produced in high concentrations (Du and Yu, 2002). Like the MMCs, complex food wastes and other inexpensive carbon sources like food scraps from restaurants or kitchens (Du and Yu, 2002; Du *et al*., 2004; Hafuka *et al*., 2011; Omar *et al*., 2011; Eshtaya *et al*., 2013) or pea shells (Patel *et al*., 2012) can be used as nutrient sources for PHA production with minimal pre‐treatment steps.

One of the main difficulties of the indirect coupling process is efficiently harvesting the VFAs and transferring them to a pure culture fermenter. This was initially achieved using evaporation and ion exchange, but the processes were costly. In one of the earliest alternative approaches, Du and Yu (2002) and Du *et al*. (2004) compared using a silicone membrane with a dialysis membrane to diffuse the acids into an air‐bubbling reactor, while preventing solids from mixing. The dialysis membrane worked considerably better, allowing a maximum dry cell weight and PHBV concentration of 22.7 g l^−1^ and 72.6%, respectively, compared to a maximum of 11.3 g l^−1^ and 60.2% PHB (as opposed to the PHBV) when a silicon membrane was used. The primary problem was that the process still consumed a high amount of operational energy due to small membrane pore sizes. A subsequent study saw success with harvesting slurry once a week and filtering the VFAs using a 0.45‐μm filter (Hafuka *et al*., 2011). Another difficulty with indirect coupling is that the VFAs are inhibitory to growth in high concentrations, which means that it is often desirable to use fed‐batch approaches to keep concentrations low, while ensuring that VFAs are available for consumption (Omar *et al*., 2011). Despite these problems, using MMC to produce VFAs that are utilized by pure cultures is a way to combine many of the best aspects of pure culture and MMC production of PHAs.

## Conclusions and outlook

Utilizing food waste to create PHAs has potential for long term applications, but is not without some hurdles. In most cases, many of the food waste sources were locally procured and this is an important consideration as transportation of food waste to the source of the microbial PHA production systems could be cost prohibitive. Furthermore, the contributions of food waste pre‐treatment to overall process costs of microbial PHA synthesis need to be examined in‐depth. Ideally, pre‐treatment of food waste should be kept to a minimal to reduce time and cost. PHA extraction from microbial biomass should also be considered in a technoeconomic analysis. The most common PHA extraction methods are solvent‐based extraction; however, there are a variety of different methods that can be used (Sabirova *et al*., 2006; Madkour *et al*., 2013; Rahman *et al*., 2013). In addition, a biorefinery concept could be realized with food waste being the feedstock to producing PHAs and additional products, as has been proposed by others (Lin *et al*., 2013; Pfaltzgraff *et al*., 2013; Dietrich *et al*., 2017). Biorefineries offer the advantages of not depending on a single product to be produced and thus are flexible and potentially sustainable.

The microbes used to convert food waste to PHAs are diverse, ranging from known, well‐defined microorganisms to mixed microbial consortia. As mentioned, both natively accumulating PHA strains and genetically engineered strains could be used as platforms for PHA production. Strain selection is important aspect of PHA production and bioprospecting could lead to the discovery of additional microbes that can be used as PHA production strains in the future (Prieto, 2016). In addition to engineering microbes to produce PHAs, microbes could also be optimized to use specific food substrates and generate defined chain‐length PHAs.

Food wastage is a global issue; the ability to upgrade complex carbon substrates into a tangible product such as PHAs could help reduce the burden of waste processing by municipalities. As described here, many different routes on food waste conversion to PHAs exist. There is not a single solution to a specific type of food waste, rather there are multiple paths that could be taken, each with different pros and cons. New and innovative methods of food waste processing to PHAs will continue to grow in the future, and these emerging technologies could make the economic production of microbial PHAs a reality in the not‐to‐distant future.

## Conflict of interest

None declared.
